# Diagnostic accuracy of screening tests for COPD: a systematic review and meta-analysis

**DOI:** 10.1136/bmjopen-2015-008133

**Published:** 2015-10-08

**Authors:** Shamil Haroon, Rachel Jordan, Yemisi Takwoingi, Peymane Adab

**Affiliations:** Public Health, Epidemiology & Biostatistics, School of Health and Population Sciences, University of Birmingham, Birmingham, UK

**Keywords:** PUBLIC HEALTH, PRIMARY CARE, PREVENTIVE MEDICINE, EPIDEMIOLOGY

## Abstract

**Background:**

Chronic obstructive pulmonary disease (COPD) is widely underdiagnosed. A number of studies have evaluated the accuracy of screening tests for COPD, but their findings have not been formally summarised. We therefore sought to determine and compare the diagnostic accuracy of such screening tests in primary care.

**Methods:**

Systematic review and meta-analysis of the diagnostic accuracy of screening tests for COPD confirmed by spirometry in primary care. We searched MEDLINE, EMBASE and other bibliographic databases from 1997 to 2013 for diagnostic accuracy studies that evaluated 1 or more index tests in primary care among individuals aged ≥35 years with no prior diagnosis of COPD. Bivariate meta-analysis of sensitivity and specificity was performed where appropriate. Methodological quality was assessed independently by 2 reviewers using the QUADAS-2 tool.

**Results:**

10 studies were included. 8 assessed screening questionnaires (the COPD Diagnostic Questionnaire (CDQ) was the most evaluated, n=4), 4 assessed handheld flow meters (eg, COPD-6) and 1 assessed their combination. Among ever smokers, the CDQ (score threshold ≥19.5; n=4) had a pooled sensitivity of 64.5% (95% CI 59.9% to 68.8%) and specificity of 65.2% (52.9% to 75.8%), and handheld flow meters (n=3) had a sensitivity of 79.9% (95% CI 74.2% to 84.7%) and specificity of 84.4% (68.9% to 93.0%). Inadequate blinding between index tests and spirometry was the main risk of bias.

**Conclusions:**

Handheld flow meters demonstrated higher test accuracy than the CDQ for COPD screening in primary care. The choice of alternative screening tests within whole screening programmes should now be fully evaluated.

**PROSPERO registration number:**

CRD42012002074.

**Strengths and limitations of this study**

- This is the first systematic review and meta-analysis of the diagnostic accuracy of screening tests for chronic obstructive pulmonary disease (COPD) in primary care.
- Robust methods were used to identify, appraise and summarise the available literature.
- There were few head-to-head comparisons of screening tests.
- The definition of COPD used in the majority of included studies was physiological, based on the presence of airflow limitation, rather than clinical, requiring the presence of relevant symptoms.
- Methodological limitations of included studies included inadequate reporting of blinding of operators performing and interpreting screening and reference tests (spirometry) and reporting of withdrawals and indeterminate results.

## Introduction

Chronic obstructive pulmonary disease (COPD) is the third leading cause of death,^1^ ranks ninth for lost disability adjusted life years,^2^ and is an important cause of healthcare expenditure.^3^ Despite this, as much as 50–90% of the disease burden remains undiagnosed.^4^ Patients often under-recognise the significance of respiratory symptoms,^5^ and clinicians frequently miss opportunities to diagnose COPD at primary care consultations.^6^ Early detection may offer opportunities to reduce disease progression and improve quality of life, for example, through smoking cessation interventions^7^ and pulmonary rehabilitation.^8^ An analysis of the Health Survey for England suggested that over three-quarters of symptomatic smokers identified with COPD through targeted case finding could benefit from recommended therapies, which could potentially prevent hospitalisations.^9^

There is now a policy drive to identify undiagnosed COPD.^10^ ^11^ However, a systematic review of population-based screening with spirometry concluded that this should not be recommended, partly because it estimated that hundreds of smokers would need to be screened to prevent a single COPD exacerbation.^12^ Furthermore, without considering clinical symptoms, this approach could identify individuals with airflow obstruction, who would not meet the clinical criteria for COPD according to current guidelines.^11^

Recently, efforts to identify undiagnosed COPD have focused on the use of initial screening tests to identify those at high risk, prior to diagnostic spirometry.^13^ ^14^ Several approaches for initial screening have been evaluated, but their findings have not been systematically reviewed and quantitatively synthesised, and it is not yet clear which test or combination is the most accurate. Although one narrative review^15^ compared existing symptom-based questionnaires, it did not include other screening tests and needs updating.

We report a systematic review and meta-analysis of published studies that summarises and compares the accuracy of screening tests for COPD in primary care.

## Methods

### Protocol and registration

The protocol for this review was previously published^16^ and registered.^17^

### Eligibility criteria

We sought diagnostic accuracy studies of any design that evaluated one or more index tests, were conducted in primary care (including general practices and community pharmacies) and recruited individuals aged ≥35 years with no prior diagnosis of COPD. Index tests included screening questionnaires, handheld flow meters (eg, Piko-6 or COPD-6), peak flow meters, chest radiography, and risk prediction models or decision aids, either alone or in combination. We only included studies that specified the target condition as COPD, and used the presence of airflow obstruction, based on prebronchodilator or postbronchodilator spirometry as the reference standard (although postbronchodilator spirometry was considered the ideal reference standard).

### Outcomes

The primary outcome was identification of COPD. The main measures of test accuracy examined were sensitivity and specificity.

### Search strategy

We searched the following databases from March/April 2012 for the previous 15 years: MEDLINE, EMBASE, CINAHL, Cochrane Central Register of Controlled Trials and the Health Technology Database. We also performed an updated search on MEDLINE and EMBASE up to December 2013. Searches limited to the first 100 articles were also performed on Google Scholar, Turning Research into Practice, HTAi VORTAL and DogPile, and selected conference abstracts for the previous 2 years. Search terms are listed in online supplementary table S1 and included Medical Subject Heading terms and free-text synonyms for COPD, screening tests and measures of test accuracy, with no language restrictions.

### Study selection and data extraction

Titles and abstracts were screened independently by two reviewers. Relevant full-text articles were independently assessed for eligibility by two reviewers and disagreements resolved through discussion. Prespecified data were extracted from full-text articles by one reviewer and verified by a second. We extracted the number of true positives, false positives, true negatives and false negatives for construction of two-by-two contingency tables. Where these data were not provided, reported measures of test accuracy were used to derive these values.

### Risk of bias assessment

Included studies were assessed independently by two reviewers for risk of methodological bias and applicability concerns against criteria from the QUADAS-2 tool.^18^ Online supplementary table S2 shows how this was adapted for the review. Disagreements were resolved through discussion.

### Statistical analysis

Forest plots of sensitivity and specificity were constructed using Review Manager (RevMan) V.5.2 (Copenhagen: The Nordic Cochrane Centre, The Cochrane Collaboration, 2012). These plots were used to visually explore between-study variation in the diagnostic accuracy of each test. We also explored differences in population screened, screening test, diagnostic criteria and study design.

Where there was sufficient clinical and methodological homogeneity, we used the *xtmelogit* command in Stata V.13.1 (Stata-Corp, College Station, Texas, USA) to fit the bivariate model^19^ ^20^ to derive summary estimates of sensitivity and specificity and their 95% CIs. If there were fewer than four studies, we simplified the bivariate model to two univariate random effects logistic regression models for sensitivity and specificity by assuming no correlation between both measures.^21^ We used two approaches to compare the diagnostic accuracy of the screening tests. First, we used all relevant studies that evaluated one or more tests, and second we restricted the analysis to studies that made direct (head-to-head) comparisons. Where meta-analysis was possible, tests were compared by adding a covariate for test type to the bivariate model to assess whether average sensitivity and/or specificity differed between the tests. To assess the statistical significance of differences in sensitivity and specificity between tests, we compared the fit of alternative models (effect of adding or removing covariate terms from the model) by using likelihood ratio tests.

Positive and negative predictive values (PPV and NPV) were estimated from the sensitivity and specificity of each test, assuming a prevalence of undiagnosed COPD of 5.5%^9^ in a hypothetical population of 1000 patients aged ≥40 years. We estimated the number-needed-to-screen to identify one individual with COPD as the total number screened divided by the number of true positives, and the number of diagnostic assessments needed as the reciprocal of the PPV.

## Results

### Study selection

**Figure 1:**
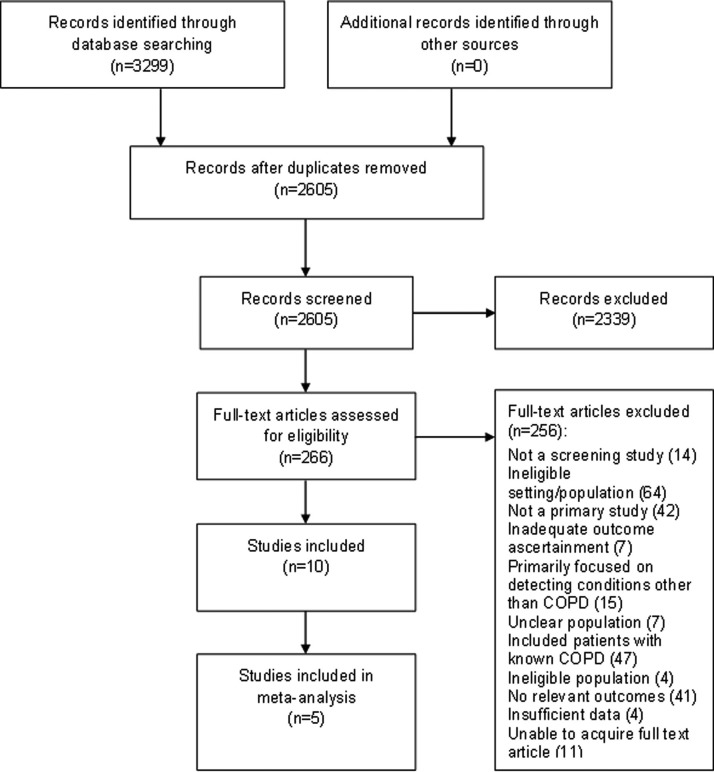
Study selection (COPD, chronic obstructive pulmonary disease).

The stages of study selection are shown in figure 1. After excluding duplicates, our search yielded 2605 records. From these, full-text articles were retrieved for 266 studies. Ten studies met the inclusion criteria, and five were suitable for meta-analysis (since these were sufficiently similar with respect to the included population, screening tests and definition of COPD). Figure 1 lists the reasons for excluding articles, the most common of which was the inclusion of patients with previously known COPD.

### Study characteristics

Characteristics of included studies are summarised in tables 1 and 2 (see online supplementary tables S3–5 for details of each study). All were cross-sectional test accuracy studies, of which two used a paired design to compare two screening tests (screening questionnaires and handheld flow meters).^22^ ^23^ Nine studies were multicentre and all were based in general practices.

**Table 1:** Characteristics of studies evaluating screening questionnaires (8 studies)^13^ ^22–25^ ^27^ ^28^ ^38^

Characteristic		Range/number of studies
Study designs	Cross-sectional test accuracy	8
Participants		237–3158
Mean age (years)		52.3–65.3
Male (%)		38.1–69.0
Required smoking status	Only current/ex-smokers	5
	Included never-smokers	3
Required respiratory symptoms		1
Setting	General practice(s)	8
Number of centres		1–36
	Multicentre	7
	Single centre	1
Recruitment strategy	Active	2
	Opportunistic	3
	Active and opportunistic	2
	Not reported	1
Questionnaires	COPD Diagnostic Questionnaire*	4
	Lung Function Questionnaire	2
	Not named	2
Common items		
	Age	7
	Smoking status	7
	Respiratory symptoms	8
	Allergies	5
Reference test—spirometry		
Post-BD		6
Definition of airflow obstruction	Post-BD FEV_1_/FVC <0.7	7
	Other†	1
Included symptoms in definition of COPD		1
Spirometry quality control	Yes	8
Range of results		
Sensitivity		57–93%
Specificity		24–80%
Severity of new COPD cases	≥80%	11–39%
(FEV_1_% predicted)‡	50–80%	43–61%
** **	<50%	10–37%

*Also referred to as the Respiratory Health Screening Questionnaire and the IPAG questionnaire.

†Pre-BD FEV_1_/FVC <88.5% predicted for men and FEV_1_/FVC <89.3% for women.

‡Based on five studies.

BD, bronchodilator; COPD, chronic obstructive pulmonary disease; FEV_1_, forced expiratory volume in 1 s; FVC, forced vital capacity; IPAG, International Primary Airways Group.

**Table 2:** Characteristics of studies evaluating handheld flow meters (4 studies)^14^ ^22^ ^23^ ^26^

Characteristic		Range/number of studies
Study designs	Cross-sectional test accuracy study	4
Participants		305–2464
Mean age (years)		52.0–65.3
Male (%)		43.3–99.7
Required smoking status	Only current/ex-smokers	3
	Included never-smokers	1
Required respiratory symptoms		0
Setting	General practice(s)	4
Number of centres		4–25
	Multicentre	4
Recruitment strategy	Active	1
	Opportunistic	2
	Active and opportunistic	1
Handheld flow meter		
Device	Piko-6	3
	COPD-6	1
Operator	Nurse	2
	GP	1
	Not reported	1
Use of BD	Pre-BD	3
	Post-BD	1
Test threshold	FEV_1_/FEV_6_<	0.70–0.75
Reference test—spirometry		
Post-BD		4
Definition of airflow obstruction	Post-BD FEV_1_/FVC <0.7	4
Included symptoms in definition of COPD		0
Spirometry quality control	Yes	2
	No	1
	Unclear	1
Range of results		
Sensitivity		79–86%
Specificity		71–99%
Severity of new COPD cases	≥80%	35–48%
(FEV_1_% predicted)^3^	50–80%	48–65%
** **	<50%	0–16%

BD, bronchodilator; COPD, chronic obstructive pulmonary disease; FEV_1_, forced expiratory volume in 1 s; FVC, forced vital capacity; GP, general practitioner.

### Recruitment and population selection

Four studies opportunistically recruited patients routinely attending primary care, three actively recruited participants through postal invitations or local advertisements, two used a combination of both strategies and one study did not report the method of recruitment.^24^ All studies specified age in the inclusion criteria with most requiring participants to be over 40 years. Seven studies also required a positive smoking history, but only one required participants to report respiratory symptoms as part of the entry criteria.^13^ The main exclusion criterion was an established history of lung disease.

### Index and reference tests

All studies first applied one or more index tests to the eligible population and then performed the reference test (spirometry) on either all (n=8 studies) or a random sample^25^ ^26^ (n=2) of participants. Index tests included screening questions or questionnaires (n=8) and handheld flow meters (n=4). One study also assessed the combined accuracy of using a screening questionnaire sequentially with a handheld flow meter.^22^ No studies evaluating other screening tests met the inclusion criteria.

### Reference standard

Prebronchodilator and postbronchodilator spirometry was the reference standard in two^25^ ^27^ and eight studies, respectively (tables 1 and 2). Most studies sufficiently described spirometry and quality control procedures. Spirometry was performed by trained technicians (n=4), general practitioners (GPs; n=1), pulmonary physicians (n=1) and nurses (n=2), while quality control was usually performed by a respiratory specialist or physiologist who reviewed spirometry results.

### Methodological quality

**Figure 2:**
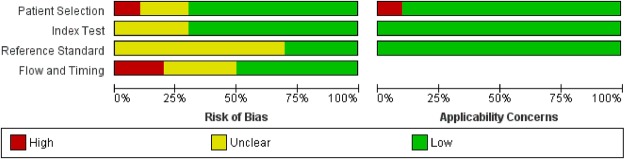
Risk of bias and applicability concerns graph: review authors’ judgements about each domain presented as percentages across included studies.

**Figure 3:**
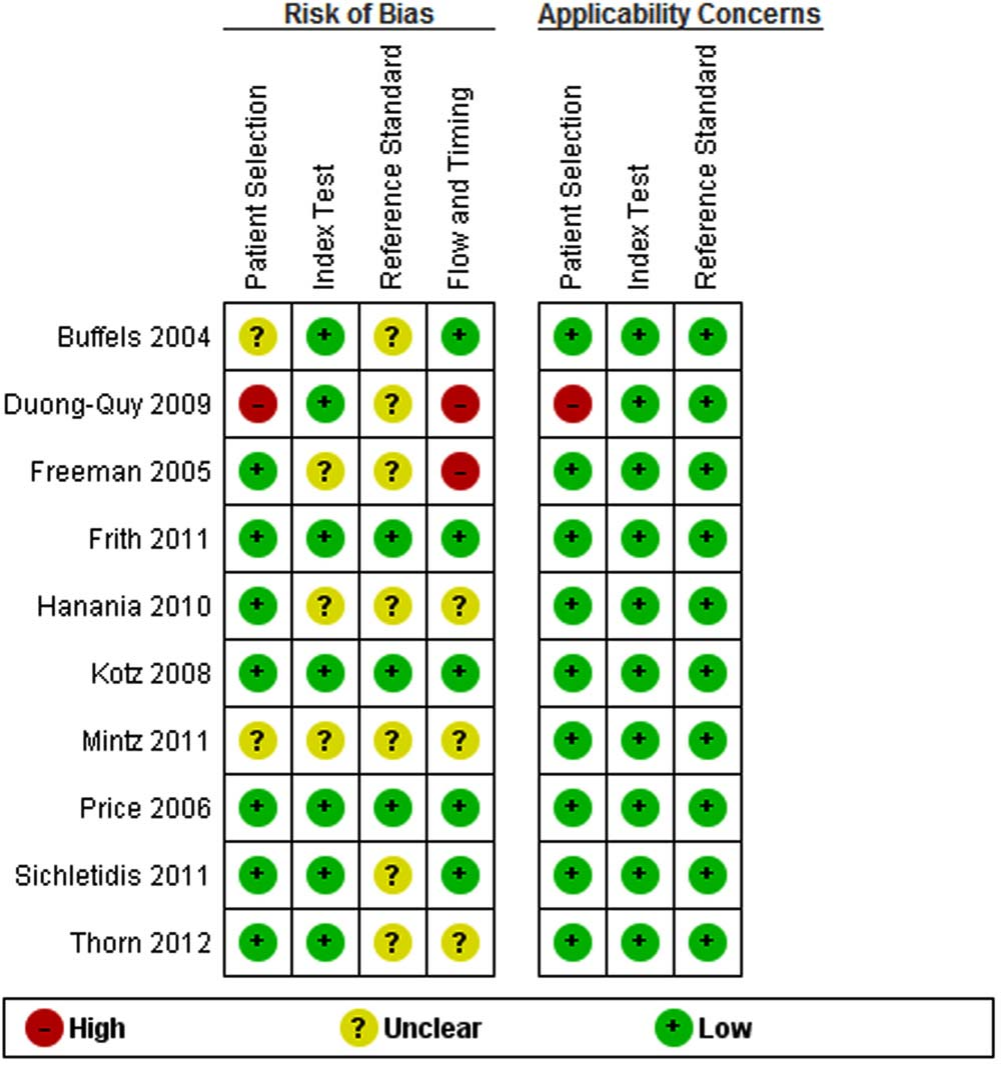
Risk of bias and applicability concerns summary: review authors’ judgements about each domain for each included study.

Most studies gave a clear description of participants, index and reference tests, and diagnostic criteria (see online supplementary figure S1 and table S6). However, there was often under-reporting of withdrawals (n=4), participant flow diagrams (n=5) and uninterpretable spirometry tests (n=5). The main risk of bias arose from inadequate blinding between index and reference tests (n=7; figures 2 and 3). There was also potential for bias in the flow and timing domain (n=5), where the number of participants undergoing index and reference tests was unclear, and where significant numbers of participants were excluded from the analysis.

### Screening questionnaires

Altogether four screening questionnaires were evaluated on a total of 9472 participants in eight studies (table 1), of which the COPD Diagnostic Questionnaire (CDQ),^28^ also referred to as the International Primary Airways Group (IPAG) Questionnaire,^22^ was the most widely validated (n=4).^13^ ^22^ ^23^ ^28^ All instruments included questions related to the presence of respiratory symptoms (usually cough, dyspnoea and wheeze). Other items included in some, but not all questionnaires related to smoking history, allergies, age, body mass index (BMI) and physical functioning. Overall, participants were similar in age (range 52.3–65.3 years) but varied by sex (range 38–69% male).

#### COPD Diagnostic Questionnaire

Four studies^13^ ^22^ ^23^ ^28^ that evaluated the CDQ in ever smokers were included in a meta-analysis. Using a score threshold of ≥19.5, the pooled sensitivity was 64.5% (95% CI 59.9% to 68.8%) and specificity 65.2% (95% CI 52.9% to 75.8%; table 3). With a prevalence of undiagnosed COPD of 5.5%, this gave a PPV of 9.7% (95% CI 6.9% to 14.2%), NPV of 96.9% (95% CI 95.8% to 97.7%), and would require 29 individuals (95% CI 27 to 31) to complete the CDQ and 11 (95% CI 7 to 15) to undergo a diagnostic assessment to identify one individual with COPD. At a lower score threshold of ≥16.5, the pooled sensitivity was higher but the specificity lower, requiring 21 individuals (95% CI 20 to 22) to complete the questionnaire and 13 (95% CI 11 to 16) to undergo a diagnostic assessment for each new diagnosis.

**Table 3:** Summary estimates of the accuracy of each test for diagnosis of COPD in ever smokers

Index test*	Studies	Cases/participants	Sensitivity (95% CI)	Specificity (95% CI)	PPV (95% CI)	NPV (95% CI)	NNS (95% CI)	NND (95% CI)
CDQ (score ≥19.5)	3	495/1703	64.5 (59.9 to 68.8)	65.2 (52.9 to 75.8)	9.7 (6.9 to 14.2)	96.9 (95.8 to 97.7)	29 (26 to 31)	11 (7 to 15)
CDQ (score ≥16.5)	4	580/2322	87.5 (83.1 to 90.9)	38.8 (27.7 to 51.3)	7.7 (6.3 to 9.8)	98.2 (96.6 to 99.0)	21 (20 to 22)	13 (11 to 16)
Handheld flow meters	3	224/1133	79.9 (74.2 to 84.7)	84.4 (68.9 to 93.0)	23.0 (12.2 to 41.3)	98.6 (97.9 to 99.1)	23 (22 to 24)	5 (3 to 9)
CDQ and handheld flow meter	1	90/624	74.4 (64.2 to 83.1)	97.0 (95.2 to 98.3)	59.1 (43.8 to 74.0)	98.5 (97.9 to 99.0)	25 (22 to 29)	2 (2 to 3)

The PPV, NPV, NNS and NND to identify one individual with COPD have been calculated assuming a prevalence of undiagnosed COPD of 5.5% in a theoretical population of 1000 people.

*Owing to the complexity of the bivariate model and the limited number of studies, only the four CDQ studies that used a score threshold ≥16.5 were pooled using a bivariate model. We carefully examined the parameter estimates of the model, especially the variances of the random effects, to check whether the model was reliable. There were only three studies of the CDQ that used a score threshold ≥19.5 and three studies of handheld flow meters. These were pooled using univariate random effects logistic regression models.

COPD, chronic obstructive pulmonary disease; CDQ, COPD Diagnostic Questionnaire; NND, number needing a diagnostic assessment to identify one with COPD; NNS, number-needed-to-screen to identify one with COPD; NPV, negative predictive value; PPV, positive predictive value.

#### All other questionnaires

**Figure 4:**
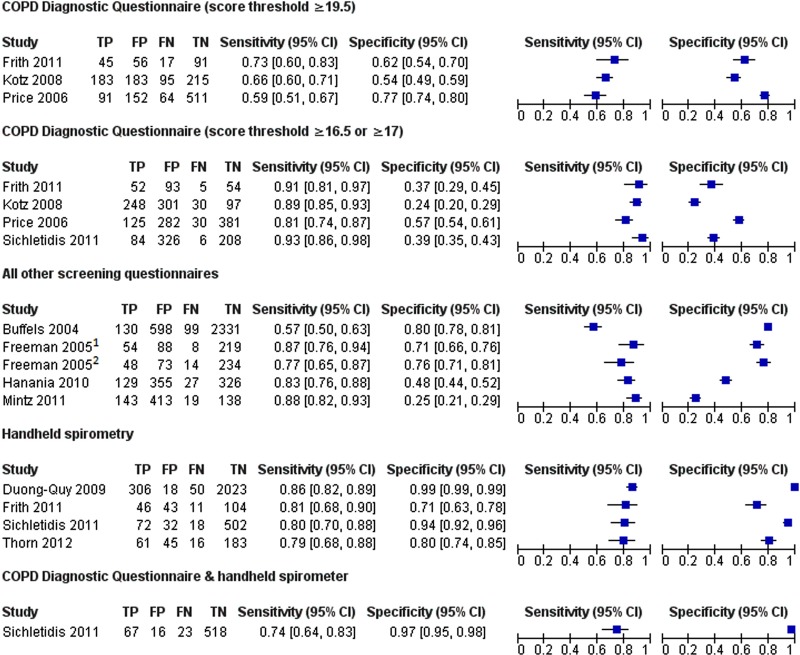
Forest plot of sensitivity and specificity of each screening test. (1) Binary response questionnaire, (2) multiple response questionnaire. COPD, chronic obstructive pulmonary disease; TP, true positive; FP, false positive; FN, false negative; TN, true negative.

There was considerable between-study heterogeneity in the design of other screening questionnaires, which precluded their meta-analysis. In these four studies, sensitivities ranged from 57% to 88% and specificities from 25% to 80% (figure 4).

### Handheld flow meters

The test accuracy of handheld flow meters was evaluated in 1400 participants across four studies (table 2).^14^ ^22^ ^23^ ^26^ Participants were similar in age (range 52–65.3 years) but varied by sex (range 43–99.7% male). Only one study included never-smokers and stratified the results by smoking status.^22^

Handheld flow meters differ from diagnostic spirometers in that they are limited to measuring the forced expiratory volume in 1 and 6 s (FEV_1_ and FEV_6_, respectively), are usually performed with three blows and are cheaper and quicker to administer. They were used without a bronchodilator in three studies^14^ ^23^ ^26^ and were supervised by either trained nurses or GPs. A narrow range of thresholds were used to denote a positive test ranging from FEV_1_/FEV_6_ <0.7 to 0.75.

Their sensitivity ranged from 79% to 86% and specificity from 71% to 99% (figure 4). Three studies^14^ ^22^ ^23^ enrolling ever smokers were similar enough to be included in a meta-analysis. The pooled sensitivity was 79.9% (95% CI 74.2% to 84.7%) and specificity was 84.4% (95% CI 68.9% to 93.0%). Using the same assumptions, this would require 23 individuals (95% CI 22 to 24) to be screened and 5 (95% CI 3 to 9) to undergo a diagnostic assessment to identify 1 individual with COPD (table 3).

### Combination of tests

**Figure 5:**
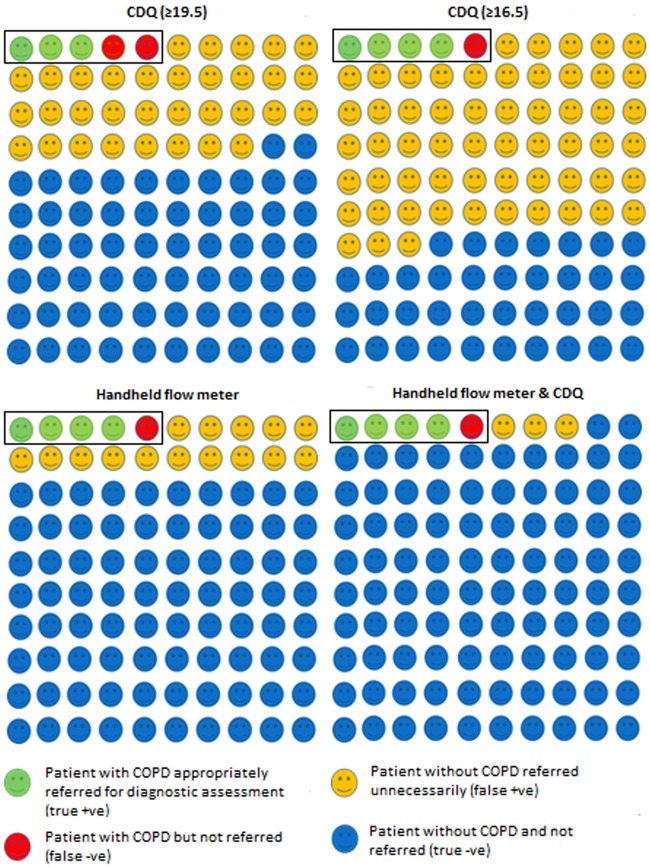
Test accuracy of each screening test in a hypothetical population of 100 participants, 5 of whom have undiagnosed COPD. COPD, chronic obstructive pulmonary disease; CDQ, COPD Diagnostic Questionnaire (score threshold).

In the single study that reported the combined accuracy of a screening questionnaire (CDQ) with a handheld flow meter, the sensitivity was 74% (95% CI 64% to 83%) and specificity was 97% (95% CI 95% to 98%).^22^ This would reduce the need for diagnostic assessment to two individuals (95% CI 2 to 3) to identify one with COPD (table 3 and figure 5).

### Comparison of test accuracy

In the first comparative analysis, based on an indirect comparison in ever smokers, there was evidence from the likelihood ratio tests that the CDQ at a score threshold of ≥19.5 had a lower sensitivity (p=0.003) but no difference in specificity (p=0.09) compared with handheld flow meters. In the second analysis at the lower score threshold of ≥16.5 (or 17), there was evidence to suggest a higher sensitivity (p=0.03) but a much lower specificity (p=0.01) than handheld flow meters. Two studies directly compared handheld flow meters and the CDQ^22^ ^23^ and their findings were consistent with the results of the indirect comparison. Furthermore, Frith *et al*^23^ also reported both higher sensitivity and specificity of handheld flow meters compared with the CDQ at the score threshold of ≥19.5.

## Discussion

### Summary of evidence

This review incorporated evidence on the test accuracy of questionnaires and handheld flow meters for COPD screening in primary care. The CDQ developed by Price *et al*^28^ was the most widely validated of the four screening questionnaires included. However, use of handheld flow meters under the supervision of trained health professionals was significantly more accurate than the CDQ for discriminating between ever smokers with and without airway obstruction, and a combination of both instruments may improve the accuracy still further, potentially reducing the number of diagnostic assessments required.^22^ Studies evaluating the CDQ and handheld flow meters had generally few methodological biases, the main being insufficient clarity on blinding between index and reference tests.

Unfortunately, only one study by Kotz *et al*^13^ considered the accuracy of a screening test (handheld flow meter) for identifying airflow obstruction in symptomatic patients, which is closer to identifying clinical COPD. The remainder evaluated the accuracy for identifying airflow obstruction without explicitly considering the presence of symptoms. Nevertheless, the results are still likely to apply since we observed that the test accuracy reported by Kotz *et al*^13^ was very similar to that reported by studies that did not explicitly consider respiratory symptoms.

### Relationship to other studies

The US Preventive Services Task Force (USPSTF) and the UK National Screening Committee recommended against routine screening for COPD partly due to concerns about efficiency and costs.^12^ ^29^ However, the USPSTF evidence review did not consider the use of screening tests such as questionnaires and handheld flow meters that may help triage high-risk patients for diagnostic assessment as suggested by our findings. Screening was recommended against on the basis that it would lead largely to the diagnosis of mild-to-moderate disease, for which there is limited evidence on effective interventions.^12^ ^30^ However, a significant proportion of new diagnoses of COPD in our included studies had moderate-to-severe airflow obstruction (48.9%^13^ to 88.5%^27^ with an FEV_1_ <80% predicted)—these patients are likely to benefit from established therapies for COPD.^8^

In 2005, van Schayck *et al*^15^ compared symptom-based questions for identifying COPD and validated their accuracy using data from the National Health and Nutrition Examination Survey (NHANES) III. Age, BMI, smoking status, smoking intensity, self-reported asthma, chronic bronchitis or emphysema, and chronic cough or phlegm represented the optimal combination of variables for identifying individuals with airflow obstruction, having a sensitivity of 71% and specificity of 67%. Many of these risk factors have been incorporated in screening questionnaires evaluated in our review and their combined accuracy appears to be lower than handheld flow meters. Furthermore, a meta-analysis of studies evaluating the accuracy of FEV_1_/FEV_6_ measured by standard diagnostic spirometry for detecting airflow obstruction showed it has a sensitivity of 0.89 (95% CI 0.83 to 0.93) and specificity of 0.98 (95% 0.95 to 0.99).^31^ While the accuracy of handheld flow meters (which measure FEV_1_/FEV_6_) appears to be lower than this, the findings from the current review suggest that they are still sufficiently accurate to screen for airflow limitation.

Finally, we identified two recent relevant studies that fell outside the time window of our literature search. The first invited ever smokers aged 40–85 years from 36 general practices to complete the CDQ and perform spirometry.^32^ The CDQ showed a sensitivity and specificity of 63.0% and 70.1%, respectively, when using a score threshold of ≥19.5 and 79.7% and 46.8% using a cut-point of ≥16.5. The second study evaluated the NPV of handheld flow meters among a small sample (n=54) of ex-smokers aged ≥50 years who had been referred for diagnostic spirometry by their GP.^33^ The NPV was estimated at 94.4% (95% CI 86.4% to 98.5%) when using the fixed ratio of FEV_1_/forced vital capacity (FVC) <0.7 to define airflow obstruction. Both findings are in keeping with our meta-analyses.

### Strengths and weaknesses of the review

Strengths of this review include the methods used to identify and appraise the available literature. Other than the limitation of the case definition discussed above, the weaknesses result mainly from the methodological limitations of included studies, particularly with respect to inadequate reporting of withdrawals and indeterminate results and blinding of operators performing and interpreting index and reference tests. This may have resulted in overestimation of test accuracy since positive index tests could plausibly influence performance and interpretation of reference spirometry. There was also a lack of head-to-head comparisons with only two studies evaluating more than one screening test.^22^ ^23^ Indirect comparisons are potentially biased because of differences in population and study characteristics.

The criteria for airflow obstruction used in the included studies is also a point of contention given that using a fixed cut-off of FEV_1_/FVC <0.7 may lead to overdiagnosis of the elderly.^34^ ^35^ Future studies should therefore consider using a definition that accounts for age, sex and ethnicity biases, ideally using an FEV_1_/FVC ratio below the lower limit of normal^36^ and using the fixed ratio for sensitivity analyses.

Finally, the included studies did not report acceptability and uptake of screening tests, which are all important for evaluating their overall effectiveness. This review can therefore only be used to comment on test accuracy and not on comparative clinical and cost-effectiveness in routine practice, which ideally should be evaluated through head-to-head trials.

### Implications for research and practice

Our findings suggest that handheld flow meters are likely to be more accurate than questionnaires for COPD screening in primary care. However, we also highlight several key limitations of previous studies. Future studies should provide clear descriptions of withdrawals, including participant flow diagrams, ensure that spirometry is performed without prior knowledge of index tests, and that indeterminate results, particularly with respect to spirometry, are reported. Future studies should also aim to recruit participants with no prior diagnosis of COPD (thus reducing the risk of spectrum bias^37^) and use a clinical case definition, rather than just airway obstruction, in order to increase generalisability to real-life practice. More studies are needed to evaluate the accuracy and effectiveness of combining screening tests and to assess their cost-effectiveness. Finally, it remains unclear whether early detection of COPD significantly improves clinical outcomes and quality of life. This should first be demonstrated in prospective studies before firm recommendations are made.

## Conclusions

Handheld flow meters used under the supervision of a trained health professional are more accurate than the CDQ for detecting spirometry-confirmed COPD in primary care. Limited evidence suggests that combining both tests may potentially improve test accuracy. Future studies should employ a case definition of COPD that aligns with current recommendations and include head-to-head comparisons.
